# Simultaneous Color Registration and Depth Completion of Point Clouds with Curriculum Learning

**DOI:** 10.3390/s25113328

**Published:** 2025-05-26

**Authors:** Juan Camilo Martinez, Ana María Montes, Cesar Marín, David Álvarez-Martínez

**Affiliations:** 1 Department of Industrial Engineering, Universidad de los Andes, Bogotá 11171, Colombia; jc.martinez10@uniandes.edu.co (J.C.M.); a.montesf@uniandes.edu.co (A.M.M.); 2 Integra S.A., Pereira 660003, Colombia; cmarin@integra.com.co

**Keywords:** depth completion, spatial transformer networks, multimodal image registration

## Abstract

Dense depth completion is critical for 3D computer vision but remains challenging when depth data are sparse and misaligned with color images due to sensor offsets. We propose a fully convolutional neural network architecture that simultaneously performs depth completion and color image registration, effectively addressing the problem of sparse depth maps and misaligned RGB inputs. Our model is trained with a novel synthetic depth generation strategy that mimics real time-of-flight (ToF) sensor noise and occlusion artifacts, helping to bridge the simulation-to-real gap. In addition, we adopt a staged curriculum learning paradigm that progressively increases task complexity over three training phases, from easy alignment scenarios to full-depth completion with simulated sensor noise. By leveraging shared features between the depth and color tasks, the joint model outperforms separate single-task approaches. At the KITTI Depth Completion benchmark, the proposed approach achieves competitive accuracy while using significantly fewer parameters and achieving faster inference than existing methods, demonstrating its effectiveness and efficiency.

## 1. Introduction

High-quality depth information is essential for accurate spatial understanding and object localization in robotics, autonomous driving, and augmented reality applications. However, obtaining dense and accurate depth maps remains challenging due to hardware limitations, occlusions, and sensor noise. Traditional depth completion methods often rely on interpolation techniques, hand-crafted features, or optimization-based formulations to infer missing depth values [1]. These classical approaches can be efficient, but they struggle to capture complex structures in highly sparse data. More recent methods leverage deep learning models—particularly convolutional neural networks (CNNs) and Transformers—to learn depth priors from large-scale datasets, achieving substantial improvements in accuracy [2]. Deep learning approaches have demonstrated the ability to handle very sparse inputs by learning from context, although they typically require extensive training data and careful network design.

A less-explored but important challenge in RGB-D sensing is the misalignment between depth sensors and color cameras. Depth sensors (such as LiDAR or stereo cameras) provide sparse point cloud data that must be projected into the camera frame, often resulting in depth maps that are not perfectly aligned with the color images due to slight differences in sensor pose or field of view. Even with calibrated intrinsic and extrinsic parameters, unmodeled distortions or baseline offsets can cause residual misalignment. This misalignment complicates the direct fusion of depth and color data, potentially leading to blurred or distorted colorized point clouds. In this work, we address simultaneous depth completion and color registration: the task of filling in sparse depth measurements while also correcting the alignment of the RGB image to the depth map coordinate frame.

In this paper, we introduce a fully convolutional neural network architecture designed to jointly perform dense depth reconstruction and color image registration. Similar multi-task ideas have been explored in related contexts—for instance, combining depth estimation and completion [3]—and have shown that shared feature learning can improve performance on each task compared to training in isolation.

Our model takes as input a sparse and potentially noisy depth map along with an RGB image that is slightly misaligned due to off-axis and off-center shifts. By leveraging shared feature learning, the network produces a dense depth map while simultaneously aligning the color image to the coordinate space of the depth map. To enhance generalization, we propose a novel synthetic data generation method that mimics real-world sensor depth sparsity and noise patterns. Prior works have demonstrated the value of synthetic data and noise modeling for depth tasks [4,5], as well as image style transfer-based domain adaptation [6], to alleviate the need for dense real-depth annotations. Our strategy builds on these insights by directly incorporating simulated noise and geometry, allowing the model to learn in a controlled yet realistic setting.

We adopt a curriculum learning strategy [7,8] to train the network in stages of increasing difficulty. Rather than exposing the model to the full complexity of the task from the start, we begin with an easier learning phase and gradually introduce harder aspects (larger misalignments, more sparse and noisy depth data). In total, we define three training stages: an initial alignment-focused stage with simplified depth representations, an intermediate stage focusing on depth completion with perfect alignment, and a final stage with all effects (noise + misalignment) combined. This staged training approach allows the network to first master simpler sub-tasks before tackling the end goal, which improves convergence and final accuracy. Curriculum learning has been shown to yield better performance in various deep learning tasks by guiding the optimization process through an organized progression of challenges [8].

We evaluate our approach on the KITTI Depth Completion benchmark [9], demonstrating competitive performance in Root Mean Square Error (RMSE) and Mean Absolute Error (MAE) compared to state-of-the-art methods.

In summary, the main contributions of this work are as follows:

- We design a novel joint depth completion and color registration network based on fully convolutional architectures, which aligns an RGB image to the depth map while completing the depth data. To our knowledge, this is one of the first approaches to address these two tasks simultaneously in a unified model.
- We propose a synthetic data generation method for training the network, using a combination of procedural noise models and 3D rendering to simulate realistic depth sensor outputs. The synthetic dataset replicates key challenges of real sensors (e.g., LiDAR or Kinect), including sparsity patterns and noise artifacts, thereby providing a rich training signal and helping to bridge the sim-to-real gap in depth completion.
- We introduce a three-stage curriculum learning paradigm tailored to the joint depth–color task. In Stage 1, the model learns to perform coarse alignment on simplified scenes with discretized depth values; in Stage 2, the model focuses on completing depth maps with increasing amounts of missing data, under perfectly aligned inputs; in Stage 3, the full task complexity is presented, including misalignments and sensor noise. This progression from easy to hard scenarios stabilizes training and leads to a more robust final model.
- We conduct extensive experiments to evaluate the real-world performance of our approach. Our joint model achieves competitive accuracy that is on par with state-of-the-art dedicated depth completion methods. Importantly, it does so with a much lower model complexity and runtime: our network has significantly fewer parameters than typical depth completion models and runs at approximately 8 Hz (0.12 s per frame) on modern GPU hardware. This efficiency makes our solution attractive for practical applications requiring real-time or embedded deployment.

The remainder of this paper is organized as follows: Section 2 reviews related work on depth completion, image alignment, and strategies for bridging the reality gap with synthetic data. Section 3 details our proposed methodology, including the neural network architecture, multi-task loss formulation, and the staged curriculum learning and data generation approach. Section 4 presents the experimental results, with discussions on training behavior, ablation studies of the curriculum, and quantitative comparisons to other methods. Finally, Section 5 concludes the paper and outlines future research directions.

## 2. Previous Work

Depth completion involves generating dense depth maps from sparse measurements, a task that varies in complexity from denoising nearly complete sensor data to inpainting missing regions in depth images and reconstructing dense maps from extremely sparse data.

### 2.1. Depth Denoising

Depth denoising focuses on eliminating noise from depth maps to enhance their accuracy. Traditional methods include filtering techniques like bilateral filtering, which smooths images while preserving edges by considering both spatial proximity and intensity differences. For example, a multiresolution bilateral filtering approach has been proposed for image denoising. Guided image filtering, another technique, utilizes a guidance image to perform edge-preserving smoothing, offering advantages over traditional methods [10]. Wavelet-based denoising methods decompose depth data into various frequency components, allowing selective noise removal from high-frequency bands while maintaining structural details. An enhanced wavelet-based method for medical image denoising, for instance, effectively removes noise while preserving critical image features [11]. Optimization-based methods, such as total variation (TV) minimization, enforce piecewise smoothness to reduce noise while preserving edges, making them effective for depth maps captured with time-of-flight (ToF) and structured-light sensors.

### 2.2. Depth Inpainting

Depth inpainting addresses the filling of missing regions in depth images, often caused by occlusions or sensor limitations. Diffusion-based approaches propagate surrounding depth values into missing areas using partial differential equations (PDEs), effectively filling small holes but potentially oversmoothing larger missing regions. Exemplar-based methods, inspired by texture synthesis techniques, improve upon diffusion-based methods by copying and adapting patches from known regions, preserving structural details more effectively. For example, an exemplar-based image inpainting algorithm has been proposed that adopts a new way of determining patch priorities and a new strategy to search the matching patches [12]. Optimization frameworks, such as low-rank matrix completion, exploit spatial correlations in the data to infer missing depth information, performing well in reconstructing planar and structured surfaces but potentially introducing errors in complex scenes. A method that improves low-rank matrix completion with low-gradient regularization has been proposed for depth image inpainting [13].

### 2.3. Depth Reconstruction

Depth reconstruction aims to generate dense depth maps from extremely sparse data, often utilizing additional guiding data like RGB images to enhance accuracy. Interpolation-based methods use different imaging modalities to guide the interpolation process, ensuring that depth discontinuities align with edges in the guiding data [14]. Deep learning approaches, particularly convolutional neural networks (CNNs), learn complex relationships between RGB images and depth maps from large datasets, often employing encoder–decoder architectures [15]. More recently, transformer-based architectures have been used, fusing RGB and depth features and using self-attention mechanisms [16,17], achieving state-of-the-art performance, albeit at a significant computational cost [2]. Some approaches that have been followed in order to reduce the computational load incurred by transformer models include knowledge distillation, where larger teacher networks are used to train smaller student models [18,19].

### 2.4. Image Registration

With the growing interest in augmented reality applications, there is a push to obtain not only geometric depth data but also color information closely aligned with the depth data to construct textured 3D elements, especially through the use of RGB-D cameras. This alignment process is known as image registration. Image registration, when applied to color and depth images, enhances the interpretability of depth data when rendered and improves performance in computer vision tasks such as object recognition and semantic segmentation, especially when extended to a three-dimensional domain. Registration algorithms are typically numerical and depend on robust estimates for intrinsic and extrinsic parameters of both depth and color cameras [4]. Even with accurate estimates, sensor geometry can introduce challenges similar to those present in depth completion. To address this problem, some authors have directed their efforts toward producing synthetic datasets through computer-generated imagery [20]. Nevertheless, a reality or sim-to-real gap exists in synthetic data.

## 3. Methodology

**Figure 1:**
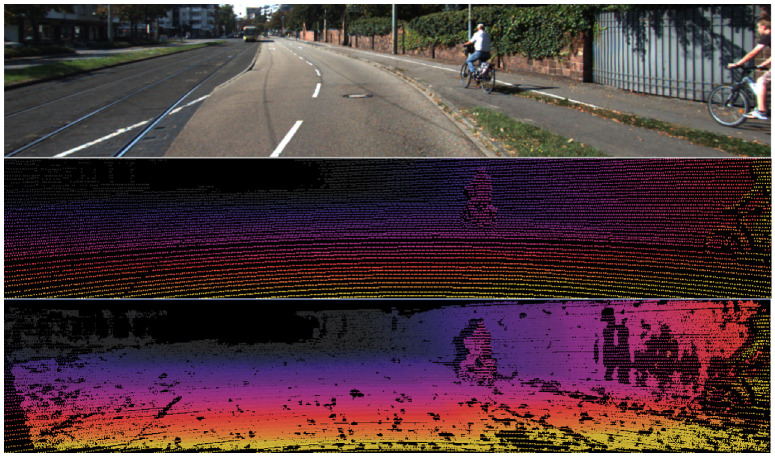
Samples of the RGB input (**top**), the sparse depth inputs (**middle**), and the dense ground truth (**bottom**).

In this work, we assume that the input data consist of two images: an RGB color image and an incomplete, sparse depth map. These images are captured using nearly coplanar sensors positioned in close proximity, ensuring minimal perspective distortion. Figure 1 illustrates examples of the RGB input, the sparse depth inputs, and the dense ground truth depth maps.

Our approach leverages a fully convolutional neural network (FCN) to simultaneously perform depth completion and color registration. The model architecture consists of two primary components:

- Registration Module: A feature alignment network estimates the transformation or optical flow required to align the color image with the depth map. For this purpose, a spatial transformer network is implemented to estimate the affine transformation between the inputs, ensuring precise alignment between modalities. We base this component on the idea of Spatial Transformer Networks [21], which provide differentiable image warping within a neural network. In our implementation, the transformer predicts an affine transformation for the RGB image, which is sufficient for the small misalignments in our setup (accounting for translation and slight scale/rotation differences).
- Depth Completion Module: This module employs a U-Net-based architecture designed to refine the sparse depth map by filling in missing regions with dense depth values. The base architecture is derived from Tiny U-Net [22], a lightweight image-to-image convolutional neural network optimized for efficiency. Compared to the original implementation, the encoder in our depth completion network includes additional skip connections on the first layer that process the concatenated 4-channel input (sparse depth plus aligned RGB, as described below), while the decoder outputs a full-resolution depth map. Skip connections from the encoder to decoder ensure that the fine-grained spatial information (such as object boundaries from the RGB image) is preserved. The use of a compact architecture is deliberate: it allows faster inference and lower memory usage, which are beneficial for real-time applications and which demonstrate that our training strategy can yield good results, even without an excessively large model.

**Figure 2:**
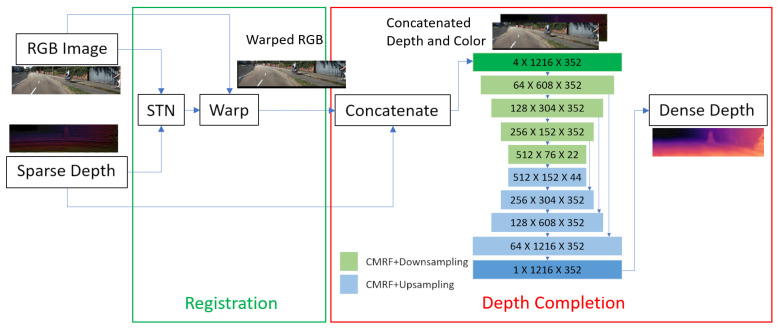
Architecture for the proposed method. Tiny U-Net blocks definitions are found in Chen’s original paper [22].

The overall architecture is depicted schematically in Figure 2. First, the RGB image and sparse depth map are passed through initial convolutional layers to extract feature maps. These feature maps are fed into the registration module, which computes the alignment warp (visualized conceptually as a flow field in the figure). The RGB feature maps (or the RGB image itself) are then warped according to this transform, producing an aligned color image. This aligned image now spatially corresponds to the depth map. We then concatenate the aligned RGB image with the original sparse depth map along the channel dimension and feed them into the depth completion module. The depth completion U-Net processes this combined input to predict the final dense depth map. By sharing the early feature extraction between the two tasks and feeding the aligned color information into depth completion, the network can use cues from the RGB (such as edges, textures, and object boundaries) to inform the depth filling process. At the same time, because the RGB is warped to depth space, the depth network does not have to struggle with misaligned features. This joint model is trained end-to-end so that the registration module and depth completion module cooperate to minimize overall error. Importantly, the training loss (described next) is multi-task: it penalizes both depth errors and any residual misalignment in color.

### 3.1. Multi-Task Loss Function

The training process employs a combined loss function, incorporating depth reconstruction loss (L1 loss) and image registration loss (SSIM loss). This multi-task learning paradigm enables information sharing between the two tasks, leading to improved accuracy in both depth completion and registration.

The L1 loss, or mean absolute error (MAE), is defined as follows:

$$L_{1}=\frac{1}{N}\sum_{i=1}^{N}\left|y_{i}-{\hat{y}}_{i}\right|$$

where

- *N* is the number of data points;
- $y_{i}$ is the true value for the *i*-th sample;
- ${\hat{y}}_{i}$ is the predicted value for the *i*-th sample.

The Structural Similarity Index (SSIM) between two images *x* and *y* is given by the following:

$$\mathrm{SSIM}\left(x,y\right)=\frac{\left(2\mu_{x}\mu_{y}+C_{1}\right)\left(2\sigma_{xy}+C_{2}\right)}{\left(\mu_{x}^{2}+\mu_{y}^{2}+C_{1}\right)\left(\sigma_{x}^{2}+\sigma_{y}^{2}+C_{2}\right)}$$

where

- $\mu_{x},\mu_{y}$ are the mean values of *x* and *y*;
- $\sigma_{x}^{2},\sigma_{y}^{2}$ are the variances of *x* and *y*;
- $\sigma_{xy}$ is the covariance between *x* and *y*;
- $C_{1}={\left(K_{1}L\right)}^{2}$ and $C_{2}={\left(K_{2}L\right)}^{2}$, where $K_{1}=0.01$, $K_{2}=0.03$, and *L* is the dynamic range of the pixel values.

The SSIM-based loss function is as follows:

$$L_{\mathrm{SSIM}}=1-\mathrm{SSIM}\left(x,y\right)$$

The aggregate loss value $L_{\mathrm{total}}$ is calculated as follows:

$$L_{\mathrm{total}}=\lambda_{1}\cdot L_{L1}+\lambda_{2}\cdot L_{\mathrm{SSIM}}$$

where

- $L_{L1}$ is the L1 loss;
- $L_{\mathrm{SSIM}}$ is the Structured Similarity Index Measure (SSIM) loss;
- $\lambda_{1}$ and $\lambda_{2}$ are the weighting factors for the L1 loss and SSIM loss, with values set to 0.9 and 0.02, respectively.

### 3.2. Curriculum Learning Strategy

To effectively train the model, we adopt a curriculum learning approach, progressively increasing task complexity across different training stages. The training data consist of synthetic scenes representing car driving environments and common household scenarios.

#### 3.2.1. Stage 1: Joint Training on Simple Scenes

**Figure 3:**
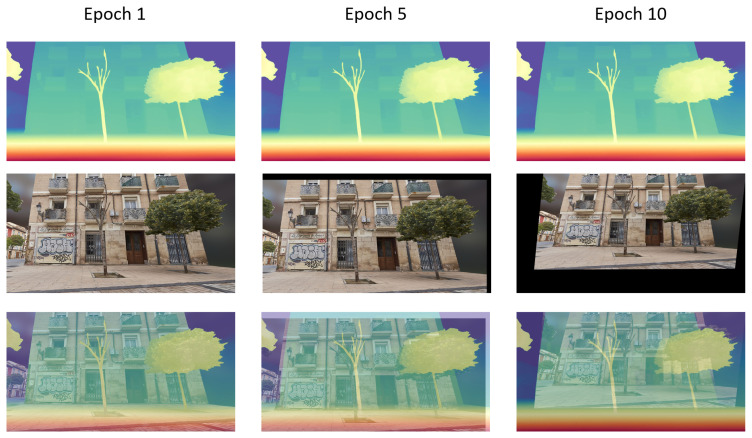
Segmentation curriculum. Each column displays progressively larger offsets between color and discretized depth images.

Initially, both modules are trained simultaneously on simple synthetic scenes designed to resemble an image segmentation task, but with class indices replaced by discretized depth values obtained via histogram binning. In total, 20 bins were considered, corresponding roughly to 6 m intervals. During this stage, the offsets between color and depth images are minimal and gradually increase in complexity. Depth completion layers are alternately frozen and unfrozen for each training epoch to encourage stable feature learning. Figure 3 illustrates this progression, with each column displaying progressively larger offsets between the color and depth images.

#### 3.2.2. Stage 2: Focused Depth Completion Training

**Figure 4:**
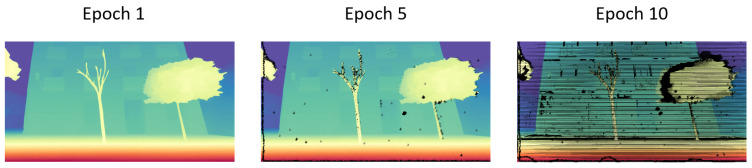
Completion curriculum. Each column shows progressively larger and more numerous missing entities.

Once the registration module achieves reasonable accuracy, its layers are frozen, and the depth completion module is trained independently. The training data for this phase consist of synthetic scenes with perfectly aligned color and depth edges, but with progressively larger patches of incomplete depth data. This ensures that the model learns to infer missing depth values effectively. Figure 4 demonstrates this curriculum, where the missing depth regions increase in size and complexity.

#### 3.2.3. Stage 3: Full Model Training on Simulated Artifacts

**Figure 5:**
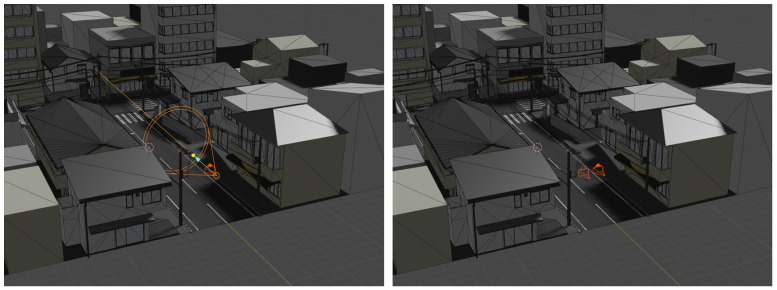
Blender scene setup for simulated TOF (**left**) and stereo imaging **(right**) sensors.

In the final training phase, all model layers are unfrozen, and the complete network is trained on image pairs designed to replicate real-world depth sensor artifacts encountered in LiDAR- and stereoscopy-based depth imaging. Synthetic depth data generation follows a combination of noise-based models [23,24] and virtual sensor simulation techniques. The latter is implemented using Blender 4.5.0 modeling software, in conjunction with the VisionBlender add-on [25], to simulate stereoscopic and time-of-flight (ToF) sensors within virtual 3D environments. Figure 5 showcases a Blender scene setup for simulated ToF (left) and stereo imaging (right) sensors.

**Figure 6:**
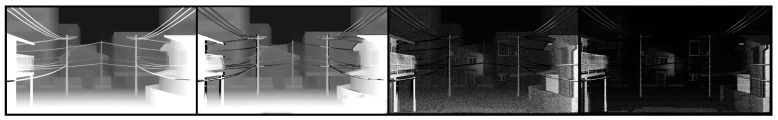
Full depth completion curriculum. Simulated depth sensor artifacts become more prevalent with every epoch.

This training strategy allows the model to progressively adapt to increasingly challenging image pairs, where geometric distortions and noise artifacts become more pervasive. Figure 6 highlights the increasing prevalence of synthetic depth sensor artifacts over successive epochs.

#### 3.2.4. Dataset and Training Details

A dataset of 6000 synthetic images is generated and split into an 80/20 train–validation distribution. The images are rendered at a resolution of 1242 × 375 pixels, matching the resolution used in the KITTI dataset. This ensures consistency with real-world benchmarks and facilitates direct comparisons with existing depth completion and registration methods.

We trained our model using PyTorch 2.2.1 on a Windows PC equipped with an Nvidia RTX 4090 GPU (24 GB VRAM), an Intel Core i7-14700 CPU, and 64 GB of RAM. Stage 1 training was run for a sufficient number of epochs to stabilize (typically 50 epochs), Stage 2 for another 50 epochs, and Stage 3 for around 100 epochs, though in practice we monitored validation loss and qualitative results to decide when to move to the next stage or stop training. We used the Adam optimizer with an initial learning rate of $1\times 10^{-3}$ in Stage 1 and Stage 2. In Stage 3, we lowered the learning rate to $5\times 10^{-5}$ and later $1\times 10^{-4}$ for fine-tuning. A small weight decay ($1\times 10^{-5}$) was applied to prevent overfitting, although overfitting risk is low given the model size and data volume. During training, we also applied simple data augmentations such as horizontal flips and slight brightness changes to the RGB images to further improve generalization. The loss evolution and training dynamics will be discussed in the next section.

## 4. Results and Discussion

### 4.1. Training

**Figure 7:**
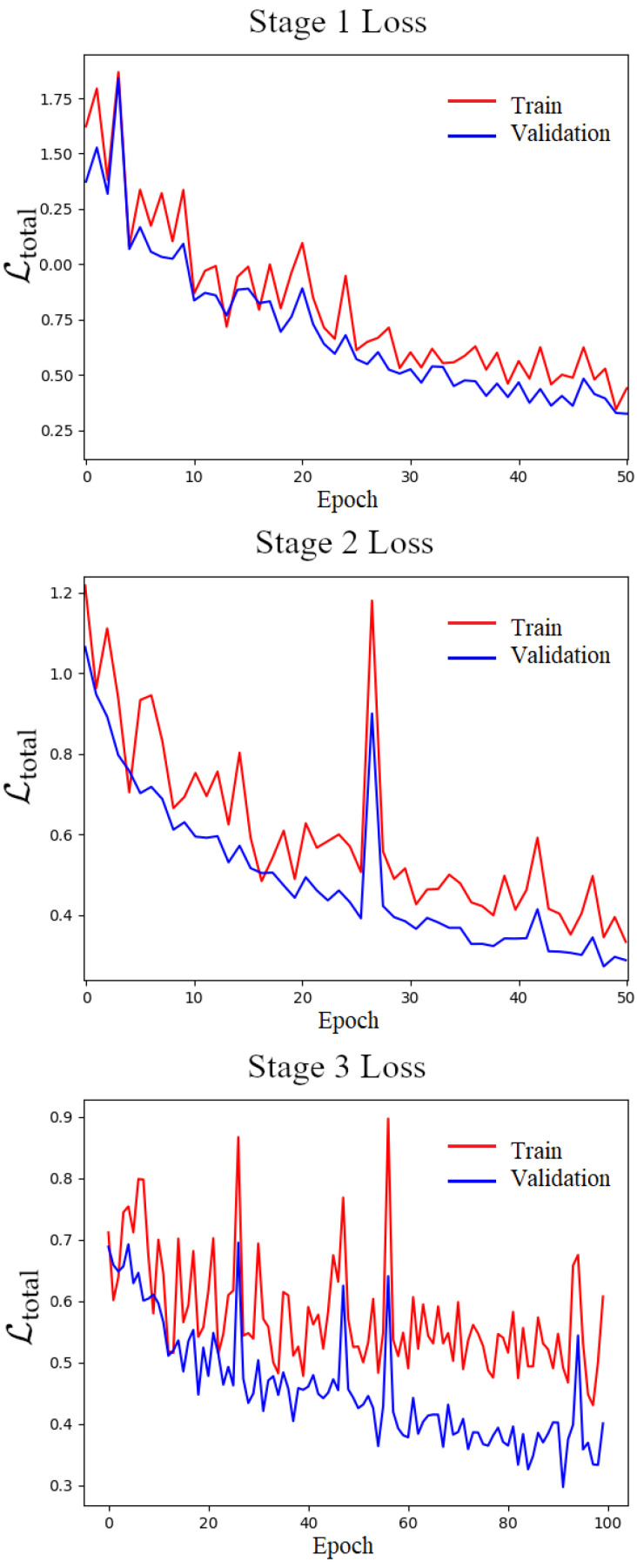
Loss evolution for Joint Training (**top**), Focused Depth Training (**middle**) and Full Model Training (**bottom**).

The training progression, as illustrated in Figure 7, exhibits a steady decrease in loss values for both the training and validation datasets. This consistent reduction in loss indicates the effectiveness of our training approach across different stages: Joint Training, Focused Depth Training, and Full Model Training.

Figure 7 illustrates the loss curves for each phase of training: the top plot corresponds to Stage 1 (Joint Training on Simple Scenes), the middle to Stage 2 (Focused Depth Completion), and the bottom to Stage 3 (Full Training with Artifacts). In Stage 1, the combined loss starts relatively high but drops rapidly within the first few epochs, indicating that the network quickly learns to align the RGB image and predict coarse depth classes. The validation loss closely follows the training loss, suggesting that the synthetic data are diverse enough to prevent overfitting in this phase. As the alignment offset increases over time (per the curriculum), we see minor upticks in the loss when a new level of difficulty is introduced, followed by continued downward trends as the model adapts.

In Stage 2, when the registration module is frozen and the task is purely depth completion on aligned inputs, the depth loss (L1) dominates. The training loss in this phase decreases steadily, and the validation loss also decreases, though sometimes at a slower rate, which is expected as the depth completion problem becomes harder (with more missing data). Importantly, the validation loss remains well above the training loss initially but converges closer by the end of Stage 2, showing that the model generalizes the inpainting capability to unseen scenes.

Stage 3 training begins with the model already reasonably good at both tasks, and now the full complexity causes an initial spike in the loss (since now the model faces the noise and misalignments that it did not see in Stage 2). However, thanks to the curriculum pretraining, the model quickly accommodates these challenges. The total loss (sum of depth and alignment terms) consistently decreases epoch over epoch in Stage 3. The gap between training and validation loss in Stage 3 is small, indicating that the model does not severely overfit to the specific artifacts in the training set—likely a benefit of using realistic but varied noise models and a large training set. By the end of training, the loss curves plateau at low values, which corresponds to high-quality qualitative results. It can be seen that loss values for subsequent stages start at a higher point than the one where convergence for the previous stage was observed. As there are certain domain differences for the synthetic data used in Stage 1 compared to Stage 2, and between synthetic and real data used for Stage 3, we presume that this is an artifact of transfer learning.

Overall, the training progression validates the effectiveness of our staged strategy: each curriculum step provided a meaningful initialization for the next, and the network avoided plateaus that we encountered when attempting to train everything at once. We noted that if we tried to train the full model from scratch on Stage 3 data (i.e., without Stages 1 and 2), the loss would often stagnate early and the model would output trivial solutions (e.g., a nearly unaltered input depth or a blurred depth map and minimal warp). In contrast, with curriculum learning, the model arrived at a good solution smoothly. This aligns with the principles of curriculum learning in deep networks, which suggest that guiding the learning process with structured data presentation can lead to better outcomes [8].

### 4.2. Ablation Study

**Figure 8:**
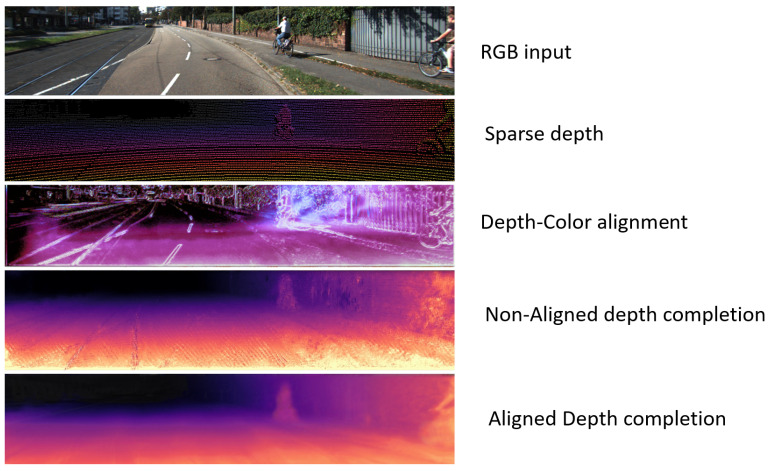
Qualitative results on synthetic validation data for each curriculum. The first row shows the input color image, the second row the sparse input depth map, and the third to fifth row the reconstructed depth maps for the different curricula.

To evaluate the impact of different training curricula, we conducted an ablation study. Figure 8 presents a qualitative comparison of the results obtained from each curriculum. The findings demonstrate that the best outcomes are achieved when the learned weights from the first two curricula are used as a starting point for the full training phase. This highlights the importance of a structured, multi-stage training process in enhancing the model’s performance.

We can clearly see the benefit of the full curriculum. The depth maps from the full curriculum have sharper object boundaries, more consistently filled surfaces, and fewer erroneous artifacts compared to the others. In contrast, the model trained without the initial alignment stage often exhibits slight ghosting or doubled edges in the depth map—an indication that misalignment was not fully corrected, causing the depth completion to blur across edges. This suggests that Stage 1 training (even on discretized depth) gave our model a head start in learning alignment that could not be easily compensated by just relying on alignment during Stage 3.

### 4.3. Quantitative Studies

Finally, to assess the real-world performance and the sim-to-real transfer capability of our approach, we evaluated the trained model on the KITTI Depth Completion benchmark [9]. KITTI provides sparse LiDAR depth maps and RGB images from a driving scenario, with dense ground truth depth for a validation set (acquired by accumulating multiple frames or using higher density scans). We used the KITTI official validation set for the quantitative evaluation of our model, as the test set requires submission to an online server (and we primarily focus on validation for this study).

The standard metrics for KITTI Depth Completion are the Root Mean Square Error (RMSE) and Mean Absolute Error (MAE) of the depth predictions, as well as their inverses (iRMSE and iMAE) which emphasize performance on farther (small depth) values. Table 1 summarizes the results of our method compared to two recent methods from the literature. The compared methods are as follows: [26], a lightweight depth completion network with knowledge distillation, and [27], an unsupervised depth completion method guided by a visual–inertial system. For each method, the table lists the iRMSE, iMAE, RMSE, and MAE on the KITTI validation set (lower is better for all metrics).

Our method achieves an RMSE of 2.14 m and MAE of 0.88 m, which is essentially on par with the state of the art. For instance, the unsupervised method [19] reports an RMSE of 2.03 and MAE of 0.89 on the validation set—a slightly lower RMSE but a slightly higher MAE than ours. Considering that [27] uses additional inertial sensor guidance and specialized training, our comparable performance is a strong result. Compared to the lightweight supervised method [26], which has RMSE 2.70 and MAE 1.00, our approach is significantly better (roughly 20% lower RMSE). Even in the inverse error metrics, our method’s iRMSE and iMAE are very competitive (780.9 and 213.5, respectively, in the units defined by KITTI), close to [27] and much better than [26]. This indicates that our model handles both near and far depth estimation reliably.

It is worth noting that our model achieves these accuracy levels while still being efficient in terms of model size and runtime. The architecture based on Tiny U-Net [22] contains far fewer parameters than typical completion models (which often use standard U-Nets or ResNet backbones). In practice, our model is about 5.2 million parameters in total—in line with the 1.17 million parameters of the model presented in [27]. For comparison, the CompletionFormer model in [17] has in the order of 30 million parameters, and even the “lightweight” model in [26] after distillation still has around 15 million. Thus, we are operating with a significantly smaller network. The inference time of our model on a single KITTI image (1242 × 375) is approximately 0.12 s on the RTX 4090 GPU. This translates to about eight frames per second, which is nearly real-time for many applications (and it could be faster on smaller resolution or with further code optimization). Many state-of-the-art methods struggle to achieve real-time inference, especially those that use heavy encoders or attention mechanisms.

The strong quantitative performance and the efficiency of our approach demonstrate that the combination of joint learning, synthetic training, and curriculum strategy is effective. The sim-to-real generalization appears to be successful; despite being trained on purely synthetic data, our model’s errors on KITTI are on par with models trained on real KITTI data (supervised) or using real sensor guidance. We attribute this success to the realism of our synthetic data (which included noise patterns akin to the Velodyne LiDAR used in KITTI) and to the robust training procedure that gradually introduced the model to the full complexity of the task. Another factor is the multi-task learning aspect—by training the network to also perform color registration, we implicitly forced it to learn more generalized features (like edge correspondences) that likely helped avoid overfitting to synthetic-specific cues and instead focus on fundamental geometric relationships that hold in real data as well.

## 5. Conclusions

In this work, we presented a novel joint learning architecture based on fully convolutional networks for simultaneous dense depth completion and color image registration. This unified approach successfully handles the challenges of incomplete depth data and misaligned color imagery by leveraging shared feature representations for both tasks.

We also introduced a synthetic depth-map generation technique that recreates realistic sensor sparsity and noise (such as ToF sensor artifacts), thereby enhancing the model’s generalization and effectively bridging the sim-to-real gap.

Furthermore, we employed a staged curriculum learning strategy to train the network, gradually increasing the alignment offset and depth sparsity in three phases to progressively build the model’s capability.

These contributions enable our model to produce accurate dense depth maps and correctly registered color images in challenging scenarios.

Notably, the proposed method attained competitive performance on the KITTI Depth Completion benchmark with a much lower model complexity—using fewer parameters and achieving near-real-time inference—compared to state-of-the-art approaches. This demonstrates that our approach delivers high accuracy and efficiency, making it a compelling solution for depth completion and image registration in resource-constrained or real-time applications.

## Figures and Tables

**Table 1:** Results comparison.

Method	RMSE	MAE	iRMSE	iMAE
[26]	891.8	238.6	2.70	1.00
[27]	771.0	214.0	2.03	0.89
Ours	780.9	213.5	2.14	0.88

## Data Availability

The original data presented in the study and all the codes are openly available in STNDepthNet at https://github.com/jcmartinez10/STNDepthNet (accessed on 1 April 2025).
